# Expression of whole blood miR-126-3p, -30a-5p, -1299, -182-5p and -30e-3p in chronic kidney disease in a South African community-based sample

**DOI:** 10.1038/s41598-022-08175-3

**Published:** 2022-03-08

**Authors:** Dipuo D. Motshwari, Cindy George, Don M. Matshazi, Cecil J. Weale, Saarah F. G. Davids, Annalise E. Zemlin, Rajiv T. Erasmus, Andre P. Kengne, Tandi E. Matsha

**Affiliations:** 1 SAMRC/CPUT/Cardiometabolic Health Research Unit, Department of Biomedical Sciences, Faculty of Health and Wellness Science, Cape Peninsula University of Technology, Cape Town, South Africa; 2 Non-Communicable Disease Research Unit, South African Medical Research Council, Parow, Francie van Zijl Drive, Parow Valley, Cape Town, South Africa; 3 Division of Chemical Pathology, Faculty of Medicine and Health Sciences, National Health Laboratory Service (NHLS) and University of Stellenbosch, Cape Town, South Africa; 4 Department of Medicine, University of Cape Town, Cape Town, South Africa

**Keywords:** Kidney diseases, Epigenetics

## Abstract

The burden of chronic kidney disease (CKD) in Africa remains poorly characterized, due partly to the lack of appropriate diagnostic strategies. Although in recent years the diagnostic and prognostic utility of microRNAs (miRNAs) have gained prominence in the context of CKD, its value has not been evaluated in African populations. We investigated the expression of whole blood miRNAs (miR-126-3p, -30a-5p, -1299, -182-5p and -30e-3p) in a total sample of 1449 comprising of 13.3% individuals with CKD (stage 1–5) and 26.4% male participants, as well as the association of these miRNAs with prevalent CKD, in a community-based sample of South African adults. We used Reverse Transcription Quantitative Real-Time PCR (RT-qPCR) to analyze miRNA expression. There was an increased expression in whole blood miR-126-3p, -30a-5p, -1299 and -182-5p in individuals with CKD, compared to those without (all p ≤ 0.036), whereas miR-30e-3p showed no significant difference between the groups (p = 0.482). Only miR-126-3p, -182-5p and -30e-3p were independently associated with increased risk of CKD (all p ≤ 0.022). This study showed for the first time that there is a dysregulation of whole blood miR-126-3p, -30a-5p, -1299 and -182-5p in South Africans of mixed-ancestry with CKD. More research is needed to ascertain their role in CKD risk screening in African populations.

## Introduction

Chronic kidney disease (CKD) is a major health concern globally, affecting approximately 8–16% of the adult population worldwide, with more than three quarters of these people residing in low-to-middle-income countries (LMICs)^1^. Although there is a paucity of data on CKD in LMICs like those in Africa, recent studies suggest an escalation in the prevalence of CKD on the African continent. Indeed, in 2014 the first systematic review on CKD prevalence reported that 13.9% of the general sub-Saharan African population had CKD^2^, and recently an increased estimated prevalence of 15.8% was reported for adults living in Africa^3^. The burden of CKD in Africa, which is partly attributable to the high prevalence of hypertension, diabetes and human immunodeficiency virus (HIV)-infection^4^, also affects younger individuals and disease progression to end-stage renal disease (ESRD) occurs at an earlier age, compared to other populations in Western countries^5^.

Currently, the diagnostic approach used to identify persons with CKD depends mainly on the estimated glomerular filtration rate (eGFR), calculated predominantly by means of the 4-variable Modification of Diet in Renal Disease (MDRD)^6^ and the Chronic Kidney Disease Epidemiology Collaboration (CKD-EPI) equation^7^. There are various limitations to the equations, for example the predominant use of serum creatinine for estimation of glomerular filtration rate which may be affected by factors such as age, sex and body mass^8^. Moreover, these prediction equations, were derived and validated in high income countries with predominately Caucasian populations. Therefore, these equations are biased and need to be validated in African populations for correct estimates of CKD burden. The limitations of these equations have stimulated interest for novel diagnostic markers with improved diagnostic/prognostic accuracy for CKD. Since their discovery in 1993^9^, microRNAs (miRNAs) have attracted immense attention in biomedical science as possible biomarkers for early detection and prognosis for various diseases such as cancer^10^, cardiovascular diseases (CVDs)^11^ and kidney diseases^12^. miRNAs are small non-coding single-stranded transcripts with approximately 19 to 24 nucleotides that regulate gene expression post transcriptionally^13^. They function by binding to the 3ʹ untranslated region (UTR) of their target messenger RNAs (mRNAs), thereby silencing gene expression either by inhibition of translation and/or by mRNA degradation^12^. To date, more than 2700 miRNAs have been identified in the human genome according to database miRbase, release 22.1^14^. A growing body of evidence suggests that miRNAs are involved in more than 90% of cellular activities, and their expression is tissue and cell type specific^15^. Furthermore, it has been found that these miRNAs are also detectable in biofluids in a highly stable manner^16^. As such, their high extracellular stability, tissue-specific expression, and feasible measurability by current techniques, makes them attractive candidate biomarkers for disease diagnosis and prognosis.

A set of miRNAs are expressed specifically in the human kidney and may be involved in the development, homeostasis, and physiology of the kidney^17^. Emerging evidence from animal models^18^ and in humans^18,19^ suggest that dysregulation of miRNAs expressed in the kidney may be associated with the development of CKD. Many of these studies quantified miRNA expression in plasma^20,21^ and serum samples^19,22^. The use of plasma or serum is subject to limitations due to a number of factors including, cell lysis bias and pre-analytical analysis which might induce contamination or variation during sample processing because of lower miRNA yield in these samples^23^.

Despite the fact that Africa has the greatest genetic diversity compared to any other region in the world^24^, there are as yet no existing studies that have examined the expression of miRNAs in African populations, thus the present study aimed: (1) to characterize the expression level of whole blood miRNAs, previously associated in the literature with kidney function or kidney disease pathophysiology, namely miR-126-3p^19^, -30a-5p^25^, -1299^26^, -182-5p^27^ and -30e-3p^28^, and (2) to investigate the association between these miRNAs and prevalent CKD, in a community-based sample of adult South Africans.

## Results

### General characteristics of the study population

The general characteristics of the participants by CKD status are summarized in Table 1. Of the 1989 individuals recruited for the VMH study, only 1449 samples were included in the present analysis. The remaining 540 samples were excluded due to missing data required to calculate eGFR. The present study comprised of 13.3% individuals with CKD (stage 1–5) and 26.4% male participants. In the group with CKD, 39.4%, 18.1%, 34.7%, 5.7% and 2.1% were in CKD stages 1 to 5, respectively. The individuals with CKD were on average older (61 vs 49 years, p < 0.0001), with a greater proportion being over the age of 60 years (53.8% vs. 21.4%, p < 0.0001). Persons with CKD further had a larger WC (96.0 vs 89.6 cm, p < 0.0001), HC (104.3 vs 101.4 cm, p = 0.001), and higher BMI (30.1 vs 27.0 kg/m^2^, p = 0.0002), compared to those without CKD. The levels of FPG, 2-h glucose, fasting insulin, 2-h insulin, HbA1c, triglycerides, total cholesterol, CRP, SBP, DBP and PP were significantly higher in participants with CKD compared to participants without CKD (all p < 0.05). Serum cotinine levels were significantly lower in persons with CKD compared to those without CKD (p = 0.0008). Individuals with CKD also had higher percentages of overweight, obese, pre-diabetic (IFD/IGT), DM and hypertension (all p < 0.0001); whereas the percentage of smokers and drinkers were significantly lower in individuals with CKD (all p < 0.0001).

**Table 1:** General characteristics of the study participants and miRNA expression by chronic kidney disease (CKD) status.

Variables	Without CKD (n = 1256)	CKD (n = 193)	p-value
Age (years)	49 (35–58)	61 (49–70)	< 0.0001
Age > 60 years (n,%)	269 (21.4)	104 (53.9)	< 0.0001
Gender (n,% male)	344 (27.4)	38 (19.7)	0.024
Weight (kg)	68.9 (57.9–84.4)	73.2 (62.2–83.3)	0.135
Waist circumference (cm)	89.6 (76.8–102.4)	96.0 (86.8–105.7)	< 0.0001
Hip circumference (cm)	101.4 (90.5–112.8)	104.3 (96.3–114.5)	0.0011
Body mass index (kg/m^2^)	27.0 (21.8–33.1)	30.1 (25.2- 34.6)	0.0002
Fasting plasma glucose (mmol/L)	4.9 (4.5–5.5)	5.3 (4.8–7.4)	< 0.0001
2-h glucose (mmol/L)	5.8 (4.7–7.3)	6.9 (5.6–8.7)	< 0.0001
Fasting insulin (IU/L)	6.5 (4.1–10.6)	7.2 (4.6–12.2)	0.015
2-h insulin (IU/L)	35.6 (18.3–66.9)	47.0 (24.4–81.4)	0.0028
Glycated haemoglobin (%)	5.7 (5.4–6.1)	6.1 (5.6–7.4)	< 0.0001
Triglycerides (mmol/L)	1.2 (0.8–1.7)	1.4 (1.0–2.0)	0.0001
HDL-C (mmol/L)	1.3 (1.1–1.5)	1.3 (1.0–1.5)	0.103
LDL-C (mmol/L)	3.0 (2.4–3.7)	3.2 (2.5–3.8)	0.105
Total cholesterol (mmol/L)	5.0 (4.3–5.8)	5.2 (4.5–6.1)	0.019
C-Reactive protein(mg/L)	3.8 (1.51–8.32)	5.1 (2.62–12.61)	< 0.0001
Serum cotinine (ng/mL)	79.7 (10–274)	10.0 (10.0–230.5)	0.0008
Systolic blood pressure (mmHg)	131 (116–149)	146 (126–165)	< 0.0001
Diastolic blood pressure (mmHg)	83 (75–93)	88 (79–100)	< 0.0001
Pulse pressure (mmHg)	70 (63–79)	73 (63–84)	0.005
**Body mass index categories (n, %)**			< 0.0001
Normal weight	501 (39.9)	48 (25.0)	
Overweight	286 (23.0)	48 (25.0)	
Obese	469 (37.7)	97 (50.5)	
**Glucose tolerance categories (n, %)**			< 0.0001
Impaired fasting glucose/impaired glucose tolerance	181 (14.4)	26 (13.5)	
Diabetes mellitus	200 (15.9)	77 (39.9)	
Hypertension (n, %)	708 (56.4)	169 (87.6)	< 0.0001
Smokers (n,%)	651 (53.9)	68 (35.4)	< 0.0001
Drinkers (n,%)	398 (31.9)	17 (8.9)	< 0.0001
miR-126-3p (2^−ΔCt^)	0.85 (0.34–1.71)	1.06 (0.38–2.35)	0.003
miR-30a-5p (2^−ΔCt^)**	0.10 (0.03–0.31)	0.15 (0.04–0.36)	0.027
miR-1299 (2^−ΔCt^)*	0.08 (0.02–0.26)	0.13 (0.02–0.46)	0.036
miR-182-5p (2^−ΔCt^)	0.93 (0.35–2.06)	1.32 (0.40–2.71)	0.005
miR-30e-3p (2^−ΔCt^)*	0.23 (0.07–0.67)	0.25 (0.06–1.00)	0.482

Data are presented as median (25th;75th percentiles) and count and percentages.

*HDL-C* high-density lipoprotein cholesterol, *LDL-C* low-density lipoprotein cholesterol), *miR* microRNA.

* and ** represent miRNAs factored by 100 and 10 respectively, as the values were very low.

### MiRNA expression

The expression levels of whole blood miRNAs, miR-126-3p, -30a-5p, -1299 and -182-5p, were significantly higher in individuals with CKD compared to those without CKD (all p ≤ 0.0364), with similar levels of miR-30e-3p observed between the two groups (p = 0.482).

### Spearman correlation coefficients

The association between circulating miRNAs (miR-126-3p, -30a-5p, -1299, -182-5p and -30e-3p), anthropometric and biochemical parameters are shown in Table 2. Only miR-126-3p was positively correlated with eGFR and ACR (r = 0.09, p = 0.045 and r = 0.10, p = 0.018, respectively). None of the other miRNAs showed an association with either measure of kidney function (p ≥ 0.063 for all). The miRNAs were also correlated with various clinical parameters. miR-126-3p, -30a-5p, -182-5p and -30e-3p were positively associated with fasting insulin (r = 0.10, p = 0.021; r = 0.10, p = 0.021; r = 0.14, p = 0.001 and r = 0.13, p = 0.004, respectively) and inversely associated with HbA1c (r =  − 0.12, p = 0.005; r =  − 0.11, p = 0.016; r =  − 0.12, p = 0.004 and r =  − 0.10, p = 0.021, respectively). Moreover, miR-126-3p showed positive correlation with WC and HC (r = 0.09, p = 0.032 and r = 0.11, p = 0.015, respectively), with miR-30a-5p positively correlated with total cholesterol (r = 0.09, p = 0.037). Higher levels of miR-182-5p were correlated with a larger HC (r = 0.09, p = 0.047). Furthermore, miR-30e-3p showed positive correlation with weight, WC and HC (r = 0.10, p = 0.024; r = 0.10, p = 0.030 and r = 0.13, p = 0.004, respectively) and negative correlation with cotinine (r =  − 0.10, p = 0.030).

**Table 2:** Spearman correlation coefficients for the association between circulating miRNAs and anthropometric and biochemical parameters.

Variables	miR-126-3p (2^−ΔCt^)		miR-30a-5p (2^−ΔCt^)		miR-1299 (2^−ΔCt^)		miR-182-5p (2^−ΔCt^)		miR-30e-3p (2^−ΔCt^)	
	r	p-value	r	p-value	r	p-value	r	p-value	r	p-value
Age (years)	− 0.02	0.588	− 0.07	0.123	0.05	0.250	− 0.03	0.493	− 0.02	0.6216
Weight (kg)	0.07	0.101	0.05	0.258	− 0.07	0.149	0.05	0.266	0.10	0.024
Waist circumference (cm)	0.09	0.032	0.05	0.216	− 0.05	0.267	0.08	0.071	0.10	0.030
Hip circumference (cm)	0.11	0.015	0.07	0.114	− 0.05	0.284	0.09	0.047	0.13	0.004
Body mass index (kg/m^2^)	− 0.02	0.423	− 0.01	0.693	− 0.02	0.412	− 0.03	0.371	0.03	0.386
Fasting plasma glucose (mmol/L)	− 0.03	0.485	− 0.02	0.661	− 0.04	0.367	− 0.03	0.516	− 0.03	0.541
2-h glucose (mmol/L)	0.02	0.690	0.01	0.761	0.05	0.291	− 0.00	0.964	0.03	0.428
Fasting insulin (IU/L)	0.10	0.021	0.10	0.021	− 0.01	0.767	0.14	0.001	0.13	0.004
2-h insulin (IU/L)	0.03	0.488	0.04	0.323	− 0.03	0.510	0.07	0.129	0.03	0.521
Glycated haemoglobin (%)	− 0.12	0.005	− 0.11	0.016	− 0.03	0.522	− 0.12	0.005	− 0.10	0.021
Triglycerides (mmol/L)	0.06	0.163	0.06	0.200	− 0.05	0.318	0.06	0.179	0.03	0.520
HDL-C (mmol/L)	0.03	0.468	0.03	0.540	− 0.01	0.869	0.00	0.961	0.01	0.859
LDL-C (mmol/L)	0.04	0.343	0.07	0.133	− 0.06	0.214	0.04	0.328	0.05	0.307
Total cholesterol (mmol/L)	0.05	0.205	0.09	0.037	-0.05	0.261	0.05	0.248	0.04	0.402
C-reactive protein (mg/L)	0.04	0.330	− 0.02	0.684	0.03	0.493	− 0.00	0.917	− 0.00	0.935
Serum cotinine (ng/mL)	− 0.03	0.491	− 0.03	0.444	0.05	0.328	− 0.04	0.337	− 0.10	0.030
Systolic blood pressure (mmHg)	0.02	0.695	0.01	0.820	0.07	0.123	− 0.03	0.512	− 0.03	0.561
Diastolic blood pressure (mmHg)	0.01	0.754	− 0.03	0.569	0.03	0.472	− 0.01	0.795	− 0.03	0.492
Pulse pressure (BPM)	0.06	0.147	− 0.02	0.604	0.05	0.277	0.05	0.211	0.05	0.223
eGFR	0.09	0.045	0.04	0.309	− 0.01	0.908	0.08	0.076	0.07	0.094
ACR	0.10	0.018	0.07	0.103	0.09	0.063	0.07	0.115	0.08	0.069

*ACR* albumin-to-creatinine ratio, *eGFR* estimated glomerular filtration rate, *HDL-C* high-density lipoprotein cholesterol, *LDL-C* low-density lipoprotein cholesterol, *miR* microRNA.

Data presented as correlation coefficient (*rho*) and p-value.

### Relationship between whole blood miRNAs and prevalent CKD

The crude and adjusted associations between whole blood miRNAs (miR-126-3p, -30a-5p, -1299, -182-5p and -30e-3p) and prevalent CKD are presented in Table 3. Higher expression of miR-126-3p, -182-5p and 30e-3p was associated with an increased prevalence of CKD (p ≤ 0.001) (Model 1), and this positive association was independent of age, sex, hypertension, DM, smoking status and drinking status (p ≤ 0.022) (Models 2–4). The miRNAs, miR-30a-5p and -1299 were not associated with prevalent CKD across all models (all p ≥ 0.093).

**Table 3:** Logistic regression analyses of whole blood miRNAs for the prediction of prevalent CKD. miR (microRNA) and OR (odds ratio).

	OR	95% Confidence interval	p-value
**miR-126-3p (2**^**−ΔCt**^**)**			
Model 1	1.33	1.19 to 1.48	< 0.0001
Model 2	1.36	1.21 to 1.52	< 0.0001
Model 3	1.34	1.20 to 1.50	< 0.0001
Model 4	1.34	1.20 to 1.51	< 0.0001
**miR-30a-5p (2**^**−ΔCt**^**)**			
Model 1	1.07	0.84 to 1.35	0.581
Model 2	1.23	0.97 to 1.57	0.093
Model 3	1.19	0.91 to 1.56	0.197
Model 4	1.20	0.91 to 1.58	0.202
**miR-1299 (2**^**−ΔCt**^**)**			
Model 1	1.10	0.98 to 1.22	0.096
Model 2	1.09	0.97 to 1.22	0.171
Model 3	1.10	0.98 to 1.23	0.095
Model 4	1.10	0.98 to 1.23	0.104
**miR-182-5p (2**^**−ΔCt**^**)**			
Model 1	1.09	1.03 to 1.15	0.001
Model 2	1.11	1.05 to 1.18	< 0.0001
Model 3	1.11	1.04 to 1.17	0.001
Model 4	1.10	1.04 to 1.17	0.001
**miR-30e-3p (2**^**−ΔCt**^**)**			
Model 1	1.18	1.02 to 1.39	0.039
Model 2	1.25	1.06 to 1.48	0.009
Model 3	1.28	1.08 to 1.52	0.005
Model 4	1.27	1.07 to 1.52	0.007

Models: Model 1: Crude; Model 2: Model 1 + age + sex; Model 3: Model 2 + hypertension + DM; Model 4: Model 3 + smoking status + drinking status.

## Discussion

The present study aimed to characterize the expression level of miR-126-3p, -30a-5p, -1299, -182-5p and -30e-3p in whole blood samples of individuals with CKD compared to those without CKD for the first time in a mixed-ancestry community in South Africa. In this cross-sectional study, we found that the expression levels of circulating miR-126-3p, 30a-5p, -1299 and -182-5p were significantly elevated in individuals with CKD. Moreover, higher levels of miR-126-3p, -182-5p and 30e-3p were independently associated with increased risk of prevalent CKD.

In accordance with our observations, a study conducted in individuals with diabetic kidney disease (DKD), showed that the plasma levels of various miRNAs, including miR-126-3p, were significantly increased in these individuals, compared to people without DKD, and this increase was positively associated with angiopoietin (Ang)-2/Ang-1 ratio, an indicator of systemic microvascular damage^21^. In line with this finding, studies have shown that the plasma level of miR-126-3p is significantly elevated in various forms of diabetes when compared to normoglycaemic controls^29,30^. The potential link between miR-126-3p and microvascular damage is further supported by a study, albeit in an animal model, which showed that vascular damage as a result of CKD is indeed associated with the upregulation of miR-126-3p in the aorta of mice^31^. Interestingly, a study by Park et al., showed that the plasma levels of miR-126-3p were significantly elevated in individuals with essential hypertension and atherosclerotic renal artery stenosis (ARAS) as compared to healthy controls^32^. Taken together, these findings and ours may suggest that increased expression of miR-126-3p is involved in disease progression and may serve as a potential marker of microvascular damage in CKD. In contrast, other studies have reported that the expression of miR-126-3p is downregulated in individuals with CKD (stages 1–5 and those on RRT)^19,22^, DKD^33^ and in individuals with ESRD^34^. In support of these reports, an animal study by Bijkerk et al., showed that overexpression of miR-126-3p in the kidneys of mice offered protection against renal ischemia by promoting vascular regeneration^35^. Also, an in vitro study on human cavernous endothelial cells (hCECs) exposed to a diabetic-like environment, showed that overexpression of miR-126-3p ameliorates endothelial dysfunction by regulating the mitogen-activated protein kinase (MAPK) signaling pathway, by reducing the expression of its target gene, Sprouty-related protein with an EVH1 domain (SPRED1)^36^. These findings^35,36^ are supported by the observation of highly expressed miR-126-3p in endothelial cells, where it is involved in processes including vascular integrity, angiogenesis and wound repair^37^.

The present study provides evidence for the first time that links miR-182-5p with an increased risk of prevalent CKD. Previous studies have shown a positive association between miR-182-5p and DKD^38^ as well as acute kidney injury (AKI)^39^. Ming et al., reported that the expression of miR-182-5p was upregulated in the podocytes of individuals with DKD, compared to non-diabetic controls. According to their findings, increased expression of miR-182-5p was associated with the downregulation of CD2-associated protein (CD2AP); which is a protein involved in podocyte apoptosis and the development of CKD in diabetics^38^. Likewise, Wilflingseder et al., found that the patients who developed AKI after kidney transplant had an increased expression level of miR-182-5p as compared to those who didn’t develop AKI post-transplant^39^. Our findings are further corroborated by the results obtained from animal studies of AKI, where inhibition of miR-182-5p in kidney tissue, improved kidney function and facilitated cell proliferation, metabolism, and angiogenesis^40^. Given that miR-182-5p is involved in a variety of processes including apoptosis, proliferation, metabolism, and angiogenesis, upregulation of this miRNA could indicate increased cell death. Taken together, these findings suggest that miR-182-5p may play a role in the pathogenesis of CKD and may serve as a potential prognostic marker and a possible therapeutic target for CKD. However, contrary to our findings, others observed that reduced expression of miR-182-5p was associated with disease development, including DKD^41^ and human renal carcinoma cells (RCC) via activation of AKT/FOXO3 signalling pathway^42^.

miR-30e-3p, which belongs to the miR-30 family (miR-30a to -e), is abundantly expressed in human glomerular podocytes cells^43^. The involvement of this family of miRNAs has been reported in processes related to podocyte injury, glomerulosclerosis, and proteinuria^43^. In the present study, we for the first time observed a positive association between miR-30e-3p and increased risk of CKD. A previous study also found plasma levels of miR-30e-3p were significantly increased, however in individuals with Contrast-Induced AKI (CI-AKI) compared to those without CI-AKI^44^. Contrary, others have reported reduced expression of miR-30e-3p in individuals with DKD^45^, with the reduced expression being associated with the transforming growth factor beta 1 (TGF-β1)-mediated epithelial–mesenchymal transition and kidney fibrosis^46^. miR-30a-5p, another member of the miR-30 family and miR-1299 showed no association with increased risk of prevalent CKD in the current study. However, their expression level was upregulated in CKD individuals as compared to those with normal kidney function. Even though the upregulation of miR-30a-5p has been reported in plasma samples of individuals with CI-AKI^44^, other studies reported the downregulation of this miRNA, in glomeruli and proximal tubules of DKD^41^ and in RCC^25^. In line with our findings, Jeong et al.^26^ also reported that miR-1299 was not associated with CKD progression. Thus, these findings taken collectively infer that miR-30-5p and -1299 may not be involved in the development of CKD.

Contradictory findings regarding the expression of miR-126-3p, miR-182-5p and miR-30e-3p in CKD from different studies may be attributable to several factors, including the differences in cohorts. Several of these studies have been evaluated in either adults of Asian or European descent, with none conducted in African populations. Studies have shown that differences in demographic factors including ethnicity^47^, age and sex^48^ influence the expression of certain miRNAs. Moreover, in the present study we included individuals from an industrialized area and even though we adjusted for smoking, we did not consider the effects of environmental factors such as air pollution. In a review by Vrijens et al., the expression of certain miRNAs were found to change as a result of exposure to smoking and air pollution^49^. Another contributing factor may have been due to the varying sample types used to quantify miRNA expression. In the present study we quantified miRNA expression in whole blood samples which comprise different blood cell populations; potentially confounding the true miRNA profile specific for CKD^50^. However, whole blood samples are superior to plasma or serum samples in that it has a high miRNA yield and is not affected by pre-analytical analysis or cell lysis bias and therefore the results may be more reliable^23^. A study by Pascut et al., showed that there were more miRNAs expressed in whole blood than serum^51^. Additionally, others have reported that certain miRNAs are highly expressed in serum as compared to plasma, which may be as a result of RNA molecules released during coagulation in serum^52^. Thus, these findings suggest that results of miRNA expressions quantified in different blood fractions are not necessarily comparable. Moreover, the lack of a validated normalization control remains a major challenge that affects the interpretability of results and may partly be the reason behind the variations and lack of comparability between studies^53^. Furthermore, some studies included individuals who were on medication, although there is evidence that corticosteroids^54^ reduce whereas metformin^55^ increases the expression of certain miRNAs. In the present study we did not adjust for any medication taken and steroids were not measured, as this was beyond the scope of our study. As such, we cannot rule out their possible effect on the observed miRNA expressions. Furthermore, our study has a high prevalence of DM, hypertension and obesity and multiple studies have shown that these conditions are independently associated with circulatory miRNAs^30,32,37^. However, this is unlikely to be the reason for the lack of comparability between the current study and others as we have adjusted for these variations. While some studies have shown that miRNA expression decreased with the severity of kidney disease^19,20^, we were unable to verify this due to the small sample size of CKD individuals which prevented stratification into the various stages of CKD. Therefore, further mechanistic studies are warranted for elucidation of the exact role of these whole blood miRNAs in CKD.

The present study has other limitations. The cross-sectional design of the study did not allow exploration of the causal relationship between miRNA expression and CKD. We used whole blood samples for miRNA quantification, without normalizing for different blood cell populations. The majority of the individuals were females; although this is a common observation in South African studies. Furthermore, a once-off creatinine measure was used for the estimation of GFR, although the KDIGO international guidelines suggest periodic measurements over a period of 3 months. Also, the kidney function was measured indirectly using eGFR which lacks precision and accuracy instead of the direct measurement of GFR which is more accurate. Moreover, due to the limited sample number of individuals with CKD in the present study we were unable to stratify the CKD group into various stages to see if the miRNAs could discriminate between the stages. The lack of validation of the current study’s findings in an independent sample serves as a major limitation, therefore the link observed between the studied miRNAs and CKD remain to be confirmed in future independent studies. However, a strength of the present study is CKD was classified using eGFR incorporated with ACR, as recommended by the KDIGO guidelines. Moreover, the present study included individuals in the early and advanced stages of CKD which serves as a further strength.

## Conclusion

To the best of our knowledge, the current study present evidence for the first time in an African population demonstrating dysregulation of whole blood miRNAs in CKD. Taken together, the findings of this study suggest that miR-126-3p, -182-5p and -30e-3p may be implicated in the development/progression of CKD and may serve as potential independent prognostic markers for CKD in this population. However, the findings of our study are still exploratory and the conflicting findings in literature suggest that CKD is heterogeneous, and the role of these miRNAs in CKD remains elusive. Large independent studies in various ethnic groups are warranted to validate our findings, explore molecular mechanisms underlying whole blood miRNA expression in CKD, and elucidate the potential relevance of these miRNAs as diagnostic and/or prognostic markers of CKD in African population before further exploration of their applicability in clinical settings.

## Materials and methods

### Study setting and participants

This study which was of a cross-sectional design, involved participants of mixed-ancestry recruited for the Vascular and Metabolic Health (VMH) study^56^. A total of 1989 individuals were recruited between 2014 and 2016 from the Bellville South area, which is located within the northern suburbs of Cape Town, South Africa. A detailed description of the study setting, and population has been reported previously^57^. Briefly, individuals aged 20 years or older who gave consent to genetic analysis were included in the current study. The exclusion criteria were ongoing pregnancy, acute illnesses and active communicable diseases.

### Questionnaire and physical examination

A standard questionnaire was used to gather information about the participants’ demographics. Participants were classified a “drinker” if they self-reported to regularly consume alcohol. The anthropometric measurements including body weight, height, waist circumference (WC) and hip circumferences (HC) were taken by trained personnel, using standardized methods. Body mass index (BMI) was calculated as weight in kilograms, divided by the square of height in meters (kg/m^2^). Participants were classified as normal weight, overweight and obese, if BMI was between 18.5–24.9, 25.0–29.99 and ≥ 30.0 kg/m^2^, respectively.

Blood pressure (BP) measurements were performed according to the World Health Organisation (WHO) guidelines^58^, using an automated digital BP monitor (Omron M6 Comfort-preformed Cuff Blood Pressure Monitor, Omron), with the participants sitting quietly in a relaxed position. Three BP readings, systolic blood pressure (SBP) and corresponding diastolic blood pressure (DBP), at one-minute intervals, were taken and the lowest reading was chosen as the participants’ BP. Pulse pressure (PP) was determined by subtracting the DBP from the SBP. Hypertension was defined as SBP ≥ 140 mmHg and/or DBP ≥ 90 mmHg or those on antihypertensive medication.

### Biochemical analysis

All biochemical analysis was conducted by an ISO 15189 accredited pathology practice (PathCare Laboratory, Cape Town, South Africa). The following biochemical parameters were measured in all participants: plasma glucose concentrations were measured using the enzymatic hexokinase method (Beckman AU, Beckman Coulter, South Africa), glycated haemoglobin (HbA1c) was measured using high performance liquid chromatography (HPLC) (Biorad Variant Turbo, South Africa), serum insulin was measured using a paramagnetic particle assay (Chemiluminescence), low-density lipoprotein cholesterol (LDL-C) was measured using an Enzymatic Selective Protection—Endpoint assay (Beckman AU, Beckman Coulter, South Africa), high-density lipoprotein cholesterol (HDL-C) using an Enzymatic Immuno-inhibition-Endpoint assay (Beckman AU, Beckman Coulter, South Africa), triglycerides were estimated using a glycerol phosphate oxidase in the presence of peroxidase (GPO-POD) Endpoint assay (Beckman AU, Beckman Coulter, South Africa), ultrasensitive C-reactive protein (CRP) was measured by Latex Particle immunoturbidimetry and serum cotinine was measured by Competitive Chemiluminescent (Immulite 2000, Siemens, South Africa). Participants were classified a “current smoker”, if serum cotinine levels were > 15 ng/mL^59^. All participants, excluding those who self-reported their diabetic status (confirmed by either participant medical card record or use of diabetic medication), underwent a 75 g oral glucose tolerance test (OGTT) after an overnight fast, according to WHO guidelines^60^. The OGTT glucose levels were used to group participants according to WHO criteria^61^ as: (1) normal glucose tolerance [fasting plasma glucose (FPG) < 6.1 mmol/L and 2 h postprandial glucose (2-h glucose) < 7.8 mmol/L]; (2) pre-diabetes including impaired fasting glucose (IFG, 6.1 ≤ FPG < 7.0 mmol/L), impaired glucose tolerance (IGT, 7.8 < 2-h glucose < 11.1 mmol/L) and the combination of both; and (3) diabetes mellitus (DM) (FPG ≥ 7.0 mmol/L and/or 2-h glucose ≥ 11.1 mmol/L). In addition to the screen detected DM, those with a history of previously diagnosed DM were also grouped as DM.

### Classification of kidney function

Urinary albumin levels were measured using the colorimetric (using bromocresol purple) method (Beckman AU, Beckman Coulter, South Africa) and serum creatinine and urinary creatinine were measured by the modified Jaffe-Kinetic method (Beckman AU, Beckman Coulter, South Africa). The participants were classified into CKD stages 1–5 using eGFR incorporated with staging based on three levels of albumin-to-creatine ratio (ACR) as recommended by the international guidelines by the Kidney Disease: Improving Global Outcomes (KDIGO) 2012^6^. The GFR was estimated using the MDRD equation^6^, without the correction factor for African ethnicity. Findings were mostly similar in secondary analyses based on CKD-EPI equation estimated GFR (data not shown). CKD stages were classified as follows: without CKD (eGFR ≥ 90 mL/min per 1.73 m^2^ and ACR < 3 mg/mmol); CKD stage 1 (eGFR ≥ 90 mL/min per 1.73 m^2^ and ACR > 3 mg/mmol), CKD stage 2 (eGFR = 60–89 mL/minute per 1.73 m^2^ and ACR > 3 mg/mmol), CKD stage 3 (eGFR = 30–59 mL/minute per 1.73 m^2^), CKD stage 4 (eGFR = 15–29 mL/min per 1.73 m^2^) and CKD stage 5 (eGFR < 15 mL/min per 1.73 m^2^). As a collective, CKD was defined as an eGFR < 90 ml/min/1.73 m^2^ and/or ACR > 3 mg/mmol).

### RNA extraction

During the survey, whole blood samples were collected in Tempus RNA tube and stored at − 20 °C for miRNAs extraction and analysis. The MagMAX™ for Stabilized Blood Tubes RNA Isolation Kit was used for extraction of total RNA, including miRNAs as per manufacturer’s specifications (Life Technologies, South Africa). RNA yield and quality were determined using Nanodrop spectrophotometry (Nanodrop one C, Thermo Fisher Scientific, USA). The concentration of RNA sample was determined by measuring its absorbance at 260 nm and A260/A280 ratio was used to determine the quality of RNA. RNA samples with concentration > 20 ng/uL and 260/280 value > 1.8 were used for miRNA quantification.

### Quantitative reverse transcription PCR (qRT-PCR)

Following total RNA isolation, miRNAs were converted to cDNA before further quantitative analysis. The subsequent reverse transcription was achieved using the TaqMan™ Advanced cDNA Synthesis Kit, and in accordance with the manufacturer’s specifications (Applied Biosystems, 2015). Quantification of miRNA expression was then performed using the Taqman Advanced miRNA assay protocol as per manufacturer’s instructions, on a QuantStudio 7 Flex (Life Technologies, USA). miR-16-5p, which showed similar 2^−ΔCt^ values between study groups (p = 0.273), was used as an endogenous control. miR-16 was identified as the most stable endogenous reference gene for miRNA studies in samples derived from individuals with CKD^62^. The delta Ct (2^−ΔCt^) method was used to assess the microRNA expression level in each sample whilst the relative miRNA expression between samples was calculated using the delta delta Ct (2^−ΔΔCT^) method^63^.

### Ethics consideration

Ethical clearance for the VMH study was granted by the Research Ethics Committees of the Cape Peninsula University of Technology (CPUT) and Stellenbosch University (NHREC: REC-230 408-014 and N14/01/003, respectively). The present study was separately approved by the CPUT Faculty of Health and Wellness Sciences Research Ethics Committee (CPUT/HW-REC 2020/H11). Participants were informed about their rights and the procedures were fully explained in the language of their choice. Written informed consent were obtained from all participants. Permission was sought from the relevant authorities in this community. All information about the participants and aspects of the study is kept confidential. Research was carried out in accordance with the Code of Ethics of the World Medical Association (Declaration of Helsinki).

### Statistical analysis

Due to the non-Gaussian distribution of most variables, the general participants’ characteristics were presented as median (25th–75th percentiles) or count and percentages. Wilcoxon rank-sum tests (continuous variables) and chi-square tests (categorical variables) were used for comparisons between individuals with CKD and those without CKD. Spearman correlation coefficients (*rho*, r) were used to assess the association between whole blood miRNAs and anthropometric and biochemical parameters. Logistic regression models were used to analyse the ability of circulating miRNAs to predict prevalent CKD. The models used were as follows: Model 1: Crude; Model 2: Model 1 + age + sex; Model 3: Model 2 + hypertension + DM; Model 4: Model 3 + smoking status + drinking status.
